# Predictors of recent mental health service utilization among firearm‐owning US service members with high levels of psychological distress

**DOI:** 10.1111/sltb.13155

**Published:** 2024-12-12

**Authors:** Taylor R. Rodriguez, Shelby L. Bandel, Allison E. Bond, Michael D. Anestis, Joye C. Anestis

**Affiliations:** ^1^ The New Jersey Gun Violence Research Center Rutgers University Piscataway New Jersey USA; ^2^ Department of Psychology Rutgers University Piscataway New Jersey USA; ^3^ Department of Urban‐Global Public Health, School of Public Health Rutgers University Piscataway New Jersey USA; ^4^ Department of Health Behavior, Society, & Policy, School of Public Health Rutgers University Piscataway New Jersey USA

**Keywords:** mental healthcare, military, service members, suicide, treatment seeking

## Abstract

**Introduction:**

Service members with mental health difficulties and access to a firearm are at an increased risk for suicide. Mental healthcare providers are well‐positioned to discuss firearms and create safety plans; however, many service members do not seek treatment. This study aims to identify potential sociodemographic predictors of recent mental healthcare utilization among firearm‐owning service members who report past month distress.

**Methods:**

The sample included 268 US military service members. Participants reported whether they attended at least one behavioral health visit in the 3 months prior to participation.

**Results:**

Females, individuals of a racial background other than Black or White, older individuals, and those who have never been active‐duty were more likely to have attended a session. Additionally, the likelihood of utilization was higher among those who reported past week wish to die and suicidal behaviors in the past year.

**Conclusion:**

While certain service members are less likely to have utilized mental healthcare, findings suggest that those with suicidal ideation and access to a firearm are likely to engage in at least one appointment. As such, providing mental healthcare providers with training and resources for promoting secure firearm storage is an important avenue for suicide prevention.

## INTRODUCTION

It has been well‐established that having access to a firearm increases the risk for dying by suicide (Studdert et al., 2020). Overall, 32.0% of the United States (US) population owns a firearm (National Firearms Survey, 2022), and firearm ownership is more common among service members and veterans. Specifically, 44.9% of veterans own a firearm (Cleveland et al., 2017). Firearms are the most common method for suicide, as approximately 55% of suicide deaths among the general population and 67.1% active‐duty military members involve a firearm (Centers for Disease Control and Prevention, 2021; Department of Defense, 2021a); highlighting that firearm suicide deaths are a major concern among the total US population and especially among military populations.

Rates of mental health disorders are also higher among military and veteran populations. Specifically, 25% of service members and veterans meet criteria for a mental health disorder, compared to 20% of the general population (Kessler et al., 2005; Seal et al., 2007). Although rates of mental health disorders are high, only 29% of service members with mental health difficulties have utilized mental healthcare services (Hom et al., 2017) and 17.2 veterans die by suicide every day (Centers for Disease Control and Prevention, 2021). The vast majority of those who experience mental health disorders do not receive care. Similarly, a majority of individuals who die by suicide with a firearm do not interact with mental healthcare (Bond et al., 2022). Notably, in a nationally representative sample of firearm‐owning service members, those who have not recently been in mental healthcare despite experiencing recent suicidal ideation were more likely to store their firearms unsecured, emphasizing that these service members have more ready access to their firearms (Anestis et al., 2023). The combination of low treatment‐seeking behaviors and high rates of firearm access, mental health disorders and suicide is concerning and highlights the need to determine ways to increase mental health treatment seeking and access to care among the military and veteran populations. To do this, an understanding of factors that impact treatment utilization is needed.

Several demographic variables have been found to differentiate those who do and do not seek mental health treatment. Specifically, research has found that education level, sex, and race impact help‐seeking behaviors. Among the general population, higher education is associated with greater mental healthcare service utilization (Steele et al., 2007), and those with lower education levels were more likely to report unmet mental healthcare needs (Zwaanswijk et al., 2003). To our knowledge, research has yet to examine how education level may impact treatment utilization among military service members. Compared to women, men are less likely to have positive help‐seeking attitudes related to common mental health issues (Wendt & Shafer, 2016); and among both the general population and military population, men are less likely to seek help for a mental health concern (Oliver et al., 2005; Turchik et al., 2012). Additionally, individuals in minoritized racial and ethnic groups are less likely to seek mental healthcare than those who identify as White. Specifically, Black individuals (Schraufnagel et al., 2006; Yang et al., 2020) and Hispanic individuals are less likely than those who are White to seek help (Alegria et al., 2002). These disparities extend into treatment engagement. Black individuals initially report more positive attitudes toward treatment than White peers, but after actual treatment utilization report more negative attitudes toward behavioral healthcare, suggesting more negative treatment experiences among Black clients than White clients (Diala et al., 2000). Structural barriers and the lack of culturally informed treatments may impact the accessibility and continuation of treatment among racial minorities. These race‐based findings are consistent within the military population as well. For example, Asian service members are less likely to seek mental healthcare than service members from other racial backgrounds (Chu et al., 2021), and members of minoritized racial and ethnic groups report a longer time to acquire mental health treatment (Goldberg et al., 2020).

Additional variables that impact treatment‐seeking behavior are sociopolitical beliefs, socioeconomic status, and military affiliation. While there is limited literature examining the impact of political beliefs on help‐seeking, it is evident that those who identify as politically conservative endorse more stigmatizing attitudes toward mental health than those who are politically liberal, and conservative news media outlets portray a more negative view of men's mental health than other media outlets (Corrigan et al., 2003; Deluca & Yanos, 2016; Pagotto et al., 2022). These negative mental health perspectives are coupled with data demonstrating that those with conservative political affiliations are twice as likely to own a firearm than those with liberal affiliations (Parker et al., 2022). Politically conservative individuals are not only at an increased risk for firearm injuries due to high rates of ownership, but they also may be less open to seeking professional help to mitigate these risks. Lower socioeconomic status is associated with low service utilization, even when services are offered free of charge (Packness et al., 2017). Research has yet to examine how sociopolitical differences and socioeconomic status impact treatment utilization among service members specifically. In terms of military affiliation, active‐duty service members are more likely to report difficulty scheduling a mental healthcare appointment and more likely to report difficulty getting time off work compared to National Guard Members (Kim et al., 2010), which may indicate that active‐duty members are less likely to seek help than those who have more ready access to civilian mental health resources.

Suicidal thoughts and behaviors are common difficulties among military members. When having these experiences during their time in the military, about half of active‐duty service members seek professional help (56.7%; Ho et al., 2018). While there are some differences in help‐seeking across demographic groups (e.g., men are less likely to discuss these experiences; Ho et al., 2018), these data suggest that help‐seeking rates for military members who are struggling with suicidal experiences are higher than help‐seeking rates among service members with other psychological difficulties (approximately 29%; Hom et al., 2017). As such, general psychological distress alone may not be a driving factor for help‐seeking. The additional difficulties that come with suicidal experiences may encourage military members to seek services.

As can be seen in previous research, several factors impact one's decision to seek treatment for mental health problems, but the majority of the existing research has been with the general population rather than service members. Additionally, much of these efforts have not focused on the many service members who own a firearm, which increases risk of firearm‐related suicide and death, especially when coupled with mental health difficulties and a lack of treatment. The present study seeks to fill this gap by examining demographic factors that impact recent treatment utilization (i.e., last 3 months) among service members who own at least one firearm and report a high level of psychological distress as measured by an abbreviated version of the Post‐Traumatic Stress Disorder (PTSD) Checklist for *Diagnostic and Statistical Manual of Mental Disorders* (*DSM‐5*; PCL‐5). While the PCL‐5 is typically utilized for initial screening of PTSD, it is recommended to be used for this purpose in conjunction with information on an experienced trauma to confirm Criterion A within the *DSM‐5* (Weathers et al., 2013). Even among trauma‐exposed National Guard troops, there is evidence of a high percentage of false positive errors when basing PTSD diagnoses on PCL‐5 scores solely; as such, this screening tool used in isolation seems to more accurately measure generalized psychological distress rather than specific aspects of PTSD (Arbisi et al., 2012). Given these findings and our lack of information regarding potential Criterion A traumas within the present study, we conceptualize elevated scores on the abbreviated PCL‐5 as psychological distress.

Among the sample, we examined how sex, race, ethnicity, education, sociopolitical beliefs, socioeconomic status, and active‐duty military status impact treatment utilization. Based on prior literature, it is hypothesized that male sex, identifying as a racial or ethnic minority, lower education levels, conservative sociopolitical views, lower socioeconomic status, and being active duty will be associated with lower rates of treatment utilization. In addition, we investigated the additional influence that past year suicidal ideation and behaviors and lifetime suicide attempts have on treatment utilization. We expect for these experiences to be associated with higher rates of treatment utilization. Findings from this study will help better understand treatment utilization among this high‐risk population.

## METHODS

### Procedure and participants

Data for the present study is a subset of a larger data collection conducted by Ipsos and utilizing the KnowledgePanel (KP) Calibration approach (Fahimi et al., 2015). All procedures were approved by the Rutgers Institutional Review Board and the US Army Medical Research and Development Command, Office of Research Protections, Human Research Protection Office (HRPO). Initial inclusion criteria for the larger sample (*n* = 719) were current affiliation with the US military and firearm ownership (see Anestis et al., 2022 for description of data collection efforts). The sample was weighted to represent the military populations' geodemographic distributions (e.g., biological sex, race, age, census region, education, and income) from the 2021 US Census Bureau's Current Population Survey (CPS). All analyses, including data descriptives, utilize weighted data. The present analyses focus only on those who elevated the PTSD Checklist for *DSM‐5* (PCL‐5; Price et al., 2016). Based upon cutoff scores described below, 269 participants qualified for the present study. One additional participant was excluded based upon unrealistic responses (i.e., reported 99 behavioral health visits in the last 3 months).

The final sample^i^ consisted of 268 US military service members who own firearms and report high levels of psychological distress over the past month. The average age of participants was 33.49 (SD = 10.79), and most participants were male (75.6%). Regarding race, 69.2% identified as White, 20.9% as Black, 10.3% as Native American or Alaska Native, 8.1% as Asian, 3.1% as Pacific Islander, and 3.1% as another racial background. Additionally, 44% identified their ethnicity as Hispanic or Latine. Most participants completed some college (14.7%), an undergraduate degree (36.4%), or a graduate degree (30.3%). Regarding political beliefs, 17.6% identified as highly conservative, 19.3% as somewhat conservative, 34.6% as moderate, 21.4% as somewhat liberal, and 7% as highly liberal. Over half (53%) reported a household income of at least $100,000, while 15.8% reported $75,000–$99,999, 10.8% reported $50,000–$74,999, 9.5% reported $25,000–$49,999, 3.2% reported $10,000–$24,999, and 4.1% reported less than $10,000. Regarding duty status, 53% reported being active‐duty at some point in their service and 47% reported never being active‐duty.

On average, participants reported attending 2.03 behavioral health visits in the 3 months prior to data collection (SD = 2.75, range 0–17). About a third of the sample (*n* = 97, 36.1%) did not attend any sessions, while 103 (38.4%) reported attending one or two sessions. Given the limited range of number of sessions attended and our interest in mental healthcare utilization overall (rather than dosage), we opted to dichotomize the dependent variable. See Table 1 for behavioral health visit descriptive data among the groups of interest in the present study. Almost half of the participants reported experiencing suicidal ideation in the past year (41.7%). Regarding suicidal behaviors, 25.8% of the sample endorsed an aborted suicide attempt (i.e., being one step away from attempting suicide but ultimately not attempting), preparatory behavior (e.g., purchasing or gathering methods that can be utilized for suicide), or practice for an attempt (e.g., hanging a rope to see if it will support their body weight) in the past year. Additionally, 20.3% of the sample reported a suicide attempt within their lifetime. Of the 92 participants who endorsed either a suicide attempt in their lifetime or other suicidal behaviors in the past year, 32 (34.9%) endorsed both. Most of the participants reported no wish to die in the past week (71.7%) while 20% reported a weak wish to die and 9.4% reported a moderate to strong wish to die.

**TABLE 1:** Behavioral health session attendance among groups.

Variables	Attended a session	Number of sessions attended
	*n* (%)	M (SD)
Overall sample	171 (63.9%)	2.03 (2.75)
Biological sex		
Male	137 (67.5%)	2.24 (2.80)
Female	34 (52.3%)	1.41 (2.48)
Ethnicity		
Hispanic/Latine	86 (72.9%)	2.24 (2.72)
Non‐Hispanic/Latine	86 (57.0%)	1.87 (2.76)
Racial identity		
White	90 (57.0%)	1.44 (2.08)
Black	35 (67.3%)	2.64 (3.40)
Other racial identity	47 (78.3%)	3.08 (3.26)
Active‐duty		
Yes	86 (60%)	1.62 (1.99)
No	85 (67.5%)	2.50 (3.35)
Suicide ideation, past year		
Yes	77 (68.8%)	2.23 (2.78)
No	94 (60.3%)	1.89 (2.72)
Suicide behaviors, past year^1^		
Yes	61 (88.4%)	3.17 (2.88)
No	110 (55.3%)	1.64 (2.59)
Suicide attempts, lifetime		
Yes	44 (80.0%)	3.05 (3.24)
No	128 (59.8%)	1.78 (2.55)

^1^ These behaviors include preparatory behavior, practice for an attempt, and aborted suicide attempts.

### Measures

#### Abbreviated post‐traumatic stress disorder checklist for DSM‐5, 8‐item version (PCL‐5; Price et al., 2016)

As previously noted, psychological distress is instantiated with the abbreviated PCL‐5 which is an 8‐item self‐report measure assessing the type and severity of PTSD‐related symptoms. Items are measured on a 4‐point Likert type scale (0 = Not at all, 4 = Extremely). The score on this measure was converted to a total PCL‐5 score in order to increase full severity ranges utilizing methods in accordance with Brier and Price (2020). Interpretive guidelines indicate that a full score of 31–33 indicates elevated symptoms (Weathers et al., 2013). Given potential error from score conversion (Brier & Price, 2020), the present study utilized a cutoff score of 33 for inclusion. Internal consistency reliability for the scale within a sample of Veteran Affairs patients seeking psychotherapy was 0.90 (Price et al., 2016). Reliability was also acceptable for the current sample of distressed firearm‐owning service members (*α* = 0.73).

#### Demographics, behavioral health visits, and wish to die

Self‐report demographics questions included biological sex, ethnicity, race, age, highest education attained, and household income. Participants also reported whether they were ever active‐duty during their military service (yes or no). Political beliefs were assessed with a 5‐point Likert‐type question with higher scores indicating more liberal beliefs (1 = highly conservative, 5 = highly liberal). Participants self‐reported the quantity of behavioral health visits they attended in the 3 months prior to data collection. Participants also reported their wish to die in the week prior to data collection on a 3‐point Likert‐type scale (0 = No wish to die, 3 = Moderate to strong wish to die).

#### Self‐injurious thoughts and behaviors interview‐short form‐self report (SITBI‐SF‐SR; Nock et al., 2007)

Select questions from the SITBI‐SF‐SR, a self‐report questionnaire assessing the characteristics of various suicidal thoughts and behaviors, were included in the present analyses. The SITBI‐SF has demonstrated good convergent and discriminant validity with military service members (Stanley et al., 2023). Participants' experiences with suicidal ideation, suicide attempt preparations and practices, aborted suicide attempts, and suicide attempts were assessed. For the present analyses, suicide attempt items were assessed across the participants' lifetime while other suicidal behaviors and suicidal ideation were assessed within the past year. All variables were coded dichotomously (yes, participant endorsed the experience or no, the participant did not).

### Data analytic plan

The present study examines demographic variables and suicidal thoughts and behaviors as potential predictors of recent mental health service utilization among firearm‐owning service members experiencing significant psychological distress. A hierarchical logistic regression was conducted with a dichotomous dependent variable identifying those who attended at least one visit in the last 3 months (*n* = 171, 63.9%) and those who reported zero visits (*n* = 97, 36.1%). Demographic variables were entered into the first step of the regression: biological sex, ethnicity (Hispanic/Latine), race, age, highest education attained, political beliefs, household income, and active‐duty status within their service. Suicidal thoughts and behaviors were entered into the second step: wish to die in the past week, suicidal ideation in the past year, suicide preparations or interrupted or aborted suicide attempts in the past year, and suicide attempts within the lifetime. Given our choice to dichotomous the dependent variable and the potential for limiting power, we also conducted a hierarchical linear regression with the continuous number of behavioral health sessions attended to compare results.

## RESULTS

Prior to examining the regression results, zero order correlation coefficients between study variables and multicollinearity statistics (see Table 2) were obtained for the model to ensure that the independent variables are not overly dependent on one another. Standard benchmarks were utilized to ensure Variance Inflation Factors (VIFs) are not above 5, tolerance values are not below 0.2, and correlation coefficients are not above 0.50 (Cohen, 1977; Kim, 2019). All VIF values were below two and tolerance values were all above 0.6, indicating that there are no concerns regarding multicollinearity. Additionally, none of the independent variables were highly correlated with one another (largest correlation was 0.41 between highest education and household income).

**TABLE 2:** Intercorrelations and multicollinearity statistics for all study variables.

Study variables	1	2	3	4	5	6	7	8	9	10	11	12	13	VIF	Tolerance
Dependent variable															
Behavioral Health Sessions (1)	–	0.14	0.16	0.19	0.08	0.09	−0.03	0.07	−0.08	0.24	0.09	0.30	0.17		
Demographic variables															
Male (2)		–	−0.09	0.01	−0.02	−0.02	−0.23	−0.08	−0.01	−0.14	−0.10	0.05	0.02	1.10	0.91
Hispanic/Latine (3)			–	0.07	−0.27	−0.19	0.11	−0.16	−0.08	0.18	0.28	0.29	0.36	1.36	0.74
Race (4)				–	0.02	0.17	0.11	0.03	−0.13	−0.02	−0.14	0.03	−0.15	1.18	0.85
Age (5)					–	0.35	0.14	0.33	0.24	−0.06	−0.13	−0.16	−0.12	1.34	0.75
Education attained (6)						–	−0.02	0.41	0.26	0.03	−0.05	−0.01	−0.06	1.43	0.70
Political beliefs (7)							–	0.04	0.05	0.11	0.12	−0.07	0.01	1.15	0.87
Household income (8)								–	0.11	−0.04	−0.01	−0.08	0.06	1.32	0.76
Active‐duty (9)									–	0.10	0.07	0.03	−0.03	1.17	0.86
Suicide‐related variables															
Wish to die, past week (10)										–	0.29	0.39	0.21	1.28	0.78
Suicidal ideation, past year (11)											–	0.35	0.32	1.31	0.76
Suicide behaviors, past year^1^ (12)												–	0.38	1.49	0.67
Suicide attempts, lifetime (13)													–	1.40	0.72

*Note*: Scores above 5 indicate high multicollinearity. Tolerance scores below 0.2 indicate high multicollinearity. Correlation coefficients between independent variables above 0.5 indicate a large association and issues with multicollinearity.

Abbreviation: VIF, variance inflation factors.

^1^ These behaviors include preparatory behavior, practice for an attempt, and aborted suicide attempts.

Regression results are presented in Table 3. The overall model predicted 30% of the variance in recent attendance at a behavioral health session. Biological sex, racial identity, age, and active‐duty status emerged as significant predictors in step one. Most of these relationships are in opposite directions than hypothesized. Compared to females, males were more than twice as likely to have been in an appointment in the 3 months prior to participation (OR = 2.79, 95% CI [1.38–5.63]). Compared to White participants, those who identified as a race other than Black or White were more than twice as likely to have utilized a behavioral health service (OR = 2.68, 95% CI [1.19–6.04]). Those who were older were also more likely to have engaged recently (OR = 1.04, 95% CI [1.01–1.07]). On the contrary, consistent with hypotheses, those who were active‐duty at some point in their service were less likely to have attended a behavioral health session (OR = 0.52, 95% CI [0.28–0.96]). Participants' reported wish to die in the past week and endorsement of suicidal behaviors in the year prior emerged as significant predictors in expected directions. Specifically, as wish to die increased, the odds of having attended a behavioral health appointment increased two and a half times (OR = 2.52, 95% CI [1.38–4.60]). Additionally, those who endorsed suicide behaviors other than an attempt (e.g., preparatory behavior and aborted attempts) in the past year had an over 300% increase in the odds of attending an appointment compared to those who did not have behaviors in the past year (OR = 3.79, 95% CI [1.53–9.37]).

**TABLE 3:** Hierarchical logistic regression predicting whether participants attended a behavioral health session in the last 3 months.

Study variables	B	Wald	*p*	Odds ratio	95% CI	Model *R* ^2^
Demographic variables						
Male	1.03	8.20	<0.01	2.79	1.38–5.63	0.15
Hispanic/Latine	0.55	2.65	0.104	1.74	0.89–3.39	
Black racial identity	0.52	1.72	0.189	1.68	0.78–3.63	
Other racial identity (not Black or White)	0.98	5.61	<0.05	2.68	1.19–6.04	
Age	0.04	5.20	<0.05	1.04	1.01–1.07	
Education attained	0.07	0.19	0.667	1.07	0.78–1.47	
Political beliefs	−0.06	0.22	0.642	0.94	0.73–1.22	
Household income	0.12	1.02	0.313	1.13	0.90–1.42	
Active‐duty	−0.66	4.39	<0.05	0.52	0.28–0.96	
Suicide‐related variables						
Wish to die, past week	0.92	9.04	<0.01	2.52	1.38–4.60	0.30
Suicidal ideation, past year	0.02	0.01	0.945	1.02	0.53–1.97	
Suicide behaviors, past year^1^	1.33	8.32	<0.01	3.79	1.53–9.37	
Suicide attempts, lifetime	0.41	0.74	0.390	1.50	0.60–3.78	

*Note*: High scores on political beliefs indicate more liberal beliefs.

Abbreviation: CI, confidence interval.

^1^ These behaviors include preparatory behavior, practice for an attempt, and aborted suicide attempts.

The hierarchical linear regression predicting the quantity of behavioral health sessions attended accounted for less variance than the logistic regression model, with an *R* ^2^ of 0.19. Most of the results matched that of the logistic regression. Males (*β* = 0.132, *p* < 0.05), those of other racial identities (*β* = 0.246, *p* < 0.001), older participants (*β* = 0.196, *p* < 0.01) all attended more sessions than the comparison group. Also, in the same direction as the linear regression, those who were active‐duty attended less sessions than those who were not (*β* = −0.165, *p* < 0.01). Consistently, those with suicide behaviors in the past year attended more sessions (*β* = 0.235 *p* < 0.001). A few differences emerged. Unlike in the logistic regression, Black participants attended more sessions than White (*β* = 0.169, *p* < 0.01), those with lifetime suicide attempts attended more sessions (*β* = 0.152, *p* < 0.05), and those with a past week wish to die did not demonstrate statistically significant differences (β = −0.043, *p* = 0.495).

## DISCUSSION

The present study sought to increase our understanding of mental health treatment seeking among service members who own firearms and are experiencing distress. Results suggest that there may be differences in treatment seeking among firearm‐owning service members related to demographic characteristics. For example, female firearm‐owning service members experiencing distress were less likely to report treatment utilization relative to male firearm‐owning service members experiencing distress. Although this finding was opposite to our expectations, this may be related to the unique treatment‐seeking barriers faced by female service members. Prior research has indicated that female service members experience many of the same barriers as their male counterparts in addition to other concerns (e.g., lack of gender sensitive treatments; Godier‐McBard et al., 2023). Further, research has indicated that these concerns experienced by female service members are associated with underutilization of treatment (Godier‐McBard et al., 2023). As such, our results may reflect a more general trend where female service members are more hesitant to seek services. Given the risk associated with firearm access when suicide risk is elevated, these results suggest that more efforts to reach female service members with psychological distress is necessary.

Findings from the present study also indicated that those who identified as a race other than White or Black were over two and a half times more likely to have been in mental health treatment relative to White firearm‐owning service members. When examining predictors of the quantity of sessions, Black participants and those of other racial identities reported utilizing more behavioral health sessions. These findings are contrary to our expectations, as previous research has demonstrated that White individuals seek treatment at higher rates relative to other racial groups (McGuire & Miranda, 2008). However, such results may reflect recent changes in mental health treatment seeking, as reports now suggest that individuals who identify as two or more races are seeking mental health treatment at similar rates to their White counterparts (American Psychiatric Association, 2017). Those classified as a race other than White or Black would include individuals who identified as two or more races; therefore, our findings may be indicative of treatment seeking within this group increasing above and beyond that of White individuals. Alternatively, these results may reflect the unique demographic characteristics of our sample, such that firearm owners and service members may interact with mental health treatment differently from the general population. For example, there are notable differences between the racial distribution of the overall US and those who serve in the military. It may be that those who opt into military service hold different beliefs about seeking mental health treatment relative to their civilian counterparts with similar racial identities. For example, there may be differences in reasons for joining the US military based on racial identity that may be indicative of an overall cultural difference, which could impact treatment seeking. However, limited research to date exists on this topic, making drawing firm conclusions difficult. Given the unexpected nature of this finding, replication is needed to ensure this result is consistent, and further research is needed to better understand how cultural differences may help explain these results.

Findings from the present study suggest that there are not associations between mental health treatment utilization and political beliefs, educational attainment, and household income among firearm‐owning service members. These results are opposite to expectations given that prior research has indicated such variables may act as barriers to mental health treatment. Here again, these results may reflect the unique characteristics of our sample. For example, among service members, these traditional barriers to treatment are less impactful relative to civilian populations, and instead other barriers are more important. For example, prior research has found that stigma is one of the most reported barriers to seeking mental health treatment among service members (Sharp et al., 2015). As such, it may be that the traditional barriers to seeking treatment are less prevalent among this group and instead concerns about stigma for seeking treatment are the more important factor. Alternatively, this finding may be related to our sample's unique distributions across these variables. Most of the present sample had at least an undergraduate degree, moderate political beliefs were the most frequently endorsed option, and over half of the sample reported a household income over $100,000. It may be that these barriers to treatment are less common among our sample simply because our sample differs from the US characteristics more broadly. The set income and access to mental health treatment associated with the military may be in part eliminating the financial barrier to mental health treatment often faced by the general population. Future research to determine if this finding is consistent and to examine the role of stigma in mental health treatment seeking among firearm‐owning service members is needed.

Results also indicated that active‐duty service members were less likely to report recent mental health treatment and attended less appointments, whereas older age was associated with a greater likelihood of and quantity of treatment utilization. Prior research has indicated that service members are often hesitant to seek mental health treatment over concerns of this interfering with their career (Sharp et al., 2015). This concern might be particularly salient for activity duty service members as they are more embedded into their military career relative to non‐active members. These concerns may also explain differences found in treatment seeking by age. Specifically, 40% of service members are under 30 years old, and nearly one‐half of active‐duty service members are 25 years or younger (Department of Defense, 2021b). It may be that greater treatment utilization among those who were older is related to being in a non‐active‐duty positions, which provide an environment where treatment feels safer and like less of a career risk. In contrast, those who are younger and still holding active‐duty positions may be avoiding treatment due to concerns that receiving mental healthcare will have an impact on their military career. Efforts to normalize and destigmatize mental health treatment among activity duty service members are needed to ensure those who need treatment feel comfortable pursuing it.

This paper also highlights that among firearm‐owning service members, suicidal thoughts and behaviors (suicide plans, preparation behaviors, and aborted attempts) are associated with mental health treatment seeking. Specifically, those who reported a wish to die in the last week were more than twice as likely to have recent mental health treatment utilization despite there being no differences in the quantity of sessions utilized. Additionally, those who engaged in suicidal behaviors in the past year were nearly four times more likely to report recent mental health services. Given the elevated risk for suicide when an individual has access to a firearm, these treatment‐seeking behaviors among those experiencing suicidal thoughts and behaviors is encouraging. Mental health providers can play an important role in firearm suicide prevention by having conversations about secure firearm storage for suicide prevention; thus, firearm‐owning service members experiencing suicidality being connected to services is encouraging. These findings highlight the importance of providing training and resources to mental health providers to equip them for conversations about secure firearm storage.

Interestingly, results did not indicate that those with a lifetime history of suicide attempts were more likely to be in mental health treatment. However, a closer examination of findings indicates that 80% of those with a lifetime suicide attempt reported recent mental health treatment suggesting that those firearm‐owning service members who previously attempted suicide are seeking treatment at relatively high rates. It is likely that the null finding reflects the statistical impact that suicidal ideation and behaviors are having on the model, such that the effects of suicide attempts are nullified. In fact, the results of the linear regression do suggest that those with firearm‐owning service members who previously attempted suicide are engaging in a higher quantity of sessions. Although prior suicide attempts are associated with increased risk for future attempts, it is likely that those represented in this group are still distinct from those who are most at risk to die by suicide. Firearms are lethal upwards of 95% of the time when used in a suicide attempt (Spicer & Miller, 2000) and those who die by firearm suicide are more likely to die on their first attempt relative to those who die using another method (Bond et al., 2022). Nevertheless, given the heightened suicide risk associated with having made a prior attempt (Bostwick et al., 2016) and ready firearm access, these findings further emphasize the importance of training mental health providers in how to have effective conversations about firearm storage.

The high rates of treatment seeking among firearm‐owning service members with a history of attempting suicide may also reflect pressure from others to seek treatment following an attempt. Among services members, there is likely a strong desire to conceal thoughts of suicide due to the perceived impact this may have on one's career. Attempting suicide likely makes concealing one's experiences with these thoughts more difficult and suicide attempt survivors may find themselves being pushed by others to seek treatment.

This study is not without limitations. First, we relied on retrospective self‐reports and, as such, there may be inaccuracy on the part of participants in their ability to recall their mental health treatment utilization. We also utilized a PTSD checklist as a measure of psychological distress. While research suggests that this is a conservative approach given our use of this measure alone, future research should discern whether findings would replicate with other measures of psychological distress (e.g., Depression Anxiety Stress Scale‐21; Lovibond & Lovibond, 1995). It would also be helpful to replicate the study with a sample of trauma‐exposed military firearm owners to determine whether findings extrapolate to difficulties with PTSD. Further, we do not know what individuals consider to be mental health treatment and if this is congruent with how providers conceptualize mental health utilization. Such sampling methods also limit what type of data can be examined and as such, we do not know the nature of the services utilized among those who reported engaging in mental health services. Future research using medical records would be able to address these limitations by providing a more robust examination of what the mental health services were. Also, our sample was recruited to be representative of firearm‐owning service members; however, our selection of a subgroup of service members endorsing high levels of distress mitigates the known representativeness of the sample and limits our ability to speak to general tendencies within the US military. We were unable to examine gender differences, and future research should consider garnering data with a more diverse sample. Additionally, one recent paper found that as risk for suicidal behavior increased, firearm owners were less likely to endorse suicidal ideation (passive or active) on the SITBI (Bryan et al., 2022). As such, it may be that the SITBI and other traditional suicidal ideation measures may not capture ideation the way firearm owners experience it. Therefore, findings from the present study may not be fully capturing the samples experiences regarding suicidal ideation. Future research examining different assessments of suicidal ideation among firearm owners is necessary to ensure that we are using measures that accurately capture these experiences within this group. Finally, we do not have information about the content of individuals' mental health treatment sessions. Therefore, while we may see that some of those most at risk are in mental health treatment, it is possible that they are not disclosing their suicidal thoughts and behaviors to their clinician. Such events would inherently limit a mental health providers ability to intervene in times of suicidal crisis. Future research examining the disclosure of suicidal experiences among treatment‐seeking, firearm‐owning service members is necessary to determine if information most pertinent to suicide risk is being disclosed to providers.

Overall, this study provides valuable knowledge related to factors that are associated with mental health treatment seeking in firearm‐owning service members experiencing distress. These findings suggest that there are demographic factors and experiences with suicidality which may be associated with mental health treatment utilization. Additionally, several nonsignificant findings suggest that variables often associated with mental health treatment seeking might not be as salient within this group. Ensuring that mental health providers are trained and provided resources for conversations around secure firearm storage is paramount for suicide prevention efforts. Future research in this area is needed to determine if findings from the present study are consistent and to better understand some of the details related to mental health utilization.

## Endnote

^i^
The sample demographics were compared to the excluded participants via chi‐square and *t*‐tests. Groups did not differ based on age. However, males (*Χ* ^2^(2) = 8.80, *p* < 0.05), those who were/are active‐duty (*Χ* ^2^(2) = 6.94, *p* < 0.01), and White individuals (*Χ* ^2^(2) = 8.80, *p* < 0.01) were less likely to be included in the study. On the contrary, those of other racial backgrounds (*Χ* ^2^(2) = 10.32, *p* < 0.01) and Hispanic/Latine individuals were more likely (*Χ* ^2^(2) = 25.67, *p* < 0.001) to be included. The present sample had higher levels of education (M = 3.777, SD = 1.06) than those who were excluded (M = 3.51, SD = 1.09, *t* = −3.15, *p* < 0.01). Those in the present study reported a higher household income (M = 5.02, SD = 1.42) than those who were excluded (M = 4.82, SD = 1.30, *t* = −1.75, *p* < 0.05). Included individuals (M = 2.55, SD = 1.08) also identified as more liberal than those who were excluded (M = 2.55, SD = 1.08; *t* = −3.01, *p* < 0.01).
